# Bourgeon bronchique sarcoïdosique

**DOI:** 10.11604/pamj.2017.26.178.11763

**Published:** 2017-03-29

**Authors:** Naoual El Omri, Fadwa Mekouar

**Affiliations:** 1 Service de Médecine Interne B, Hôpital Militaire d’Instruction Mohammed V, Rabat, Maroc

**Keywords:** Sarcoïdose, bourgeon bronchique, bronchoscopie, Sarcoidosis, bronchial bud, bronchoscopy

## Image en médecine

Patiente de 53 ans, consulte pour une toux sèche et une dyspnée d’effort. Le scanner thoracique montre des adénopathies médiastino-hilaires bilatérales et interlobulaires gauches avec des atélectasies en bandes et sous segmentaires ventrales bilatérales, lingulaire et basales bilatérales. La bronchoscopie montre un aspect inflammatoire diffus et un aspect festonné de la bronche lobaire moyenne droite qui est incathéterisable avec des éperons épaissies et un bourgeon vascularisé et lisse saignant au moindre contact au niveau de la bronche segmentaire postéro-basale droite. A gauche, on trouve un bourgeon très vascularisé au niveau de la bronche postero-basale gauche de taille plus petite que celle du bourgeon droit mais il est plus inflammatoire. La biopsie montre des remaniements granulomateux en rapports avec une sarcoïdose.

**Figure 1:**
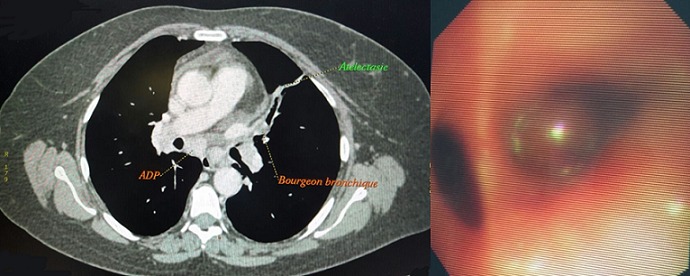
bourgeon endobronchique visible sur le TDM thoracique et à la bronchoscopie

